# Astroglial Glutamate Signaling and Uptake in the Hippocampus

**DOI:** 10.3389/fnmol.2017.00451

**Published:** 2018-01-17

**Authors:** Christine R. Rose, Lisa Felix, Andre Zeug, Dirk Dietrich, Andreas Reiner, Christian Henneberger

**Affiliations:** ^1^Institute of Neurobiology, Faculty of Mathematics and Natural Sciences, Heinrich Heine University Duesseldorf, Duesseldorf, Germany; ^2^Cellular Neurophysiology, Hannover Medical School, Hannover, Germany; ^3^Department of Neurosurgery, University of Bonn Medical School, Bonn, Germany; ^4^Cellular Neurobiology, Faculty of Biology and Biotechnology, Ruhr University Bochum, Bochum, Germany; ^5^Institute of Cellular Neurosciences, University of Bonn Medical School, Bonn, Germany; ^6^German Center for Degenerative Diseases (DZNE), Bonn, Germany; ^7^Institute of Neurology, University College London, London, United Kingdom

**Keywords:** astrocyte, glutamate receptor, glutamate transport, tripartite synapse, calcium, morphology

## Abstract

Astrocytes have long been regarded as essentially unexcitable cells that do not contribute to active signaling and information processing in the brain. Contrary to this classical view, it is now firmly established that astrocytes can specifically respond to glutamate released from neurons. Astrocyte glutamate signaling is initiated upon binding of glutamate to ionotropic and/or metabotropic receptors, which can result in calcium signaling, a major form of glial excitability. Release of so-called gliotransmitters like glutamate, ATP and D-serine from astrocytes in response to activation of glutamate receptors has been demonstrated to modulate various aspects of neuronal function in the hippocampus. In addition to receptors, glutamate binds to high-affinity, sodium-dependent transporters, which results in rapid buffering of synaptically-released glutamate, followed by its removal from the synaptic cleft through uptake into astrocytes. The degree to which astrocytes modulate and control extracellular glutamate levels through glutamate transporters depends on their expression levels and on the ionic driving forces that decrease with ongoing activity. Another major determinant of astrocytic control of glutamate levels could be the precise morphological arrangement of fine perisynaptic processes close to synapses, defining the diffusional distance for glutamate, and the spatial proximity of transporters in relation to the synaptic cleft. In this review, we will present an overview of the mechanisms and physiological role of glutamate-induced ion signaling in astrocytes in the hippocampus as mediated by receptors and transporters. Moreover, we will discuss the relevance of astroglial glutamate uptake for extracellular glutamate homeostasis, focusing on how activity-induced dynamic changes of perisynaptic processes could shape synaptic transmission at glutamatergic synapses.

## Introduction

Electrical activity of neurons is largely based on the diffusive movement of ions along their electrochemical gradient. This includes the diffusion of potassium ions (K^+^) into the extracellular space (ECS) through K^+^-permeable ion channels, resulting in a re- or hyperpolarization of the membrane potential. In addition, active neurons release chemical transmitters such as glutamate from their presynaptic terminals into the ECS. As the volume of the brain is limited, mechanisms are needed which counteract the accumulation of these substances in the ECS. A major mechanism by which this is prevented is by the cellular (re-) uptake of neurotransmitters and ions. It is well established that astrocytes take over a large part of this housekeeping function and remove molecules released by neurons from the ECS (Kofuji and Newman, 2004; Marcaggi and Attwell, 2004). Once the molecules are in the astrocytic compartment, they are either degraded, recycled or shuttled out of the brain via transport routes such as the blood or the gliolymphatic system (Abbott et al., 2006; Thrane et al., 2015).

Glutamate is the major excitatory neurotransmitter released in the brain and well known for its potential to induce excitotoxicity. At the same time, glutamate also acts as an active signaling molecule for astrocytes. This review focuses on astroglial glutamate signaling in the hippocampus, highlighting the dual role glutamate plays in the function of astrocytes. Astrocytic glutamate signaling becomes particularly interesting as these cells not only express glutamate transporters, which transport glutamate molecules into the cell with the help of ion gradients, but also several types of glutamate receptors. The relevant functional domains of sub-micron-sized astrocyte processes controlling the spread of synaptically-released glutamate is difficult to assess with current optical or electrical recording techniques. We therefore start this review with an evaluation of available tools to analyze the distribution and function of astrocytic receptors and transporters for glutamate.

Glutamate receptors on astrocytes belong to the ionotropic and metabotropic classes and are activated by synaptically released glutamate, thereby allowing astrocytes to integrate ongoing neuronal activity within a time frame in the order of tens to hundreds of milliseconds (Verkhratsky and Kirchhoff, 2007a; Bindocci et al., 2017). High-affinity glutamate transporters on astrocytes, on the other hand, represent the major mechanism for removal of glutamate at synapses and protect the brain from glutamate-induced over-excitation of neurons and excitotoxicity (Parpura et al., 2012; Schousboe et al., 2014). Like for glutamate receptors, there exist several subtypes of glutamate transporters, also named excitatory amino acid transporters (EAATs), which are differentially expressed throughout the brain and during postnatal development (Danbolt, 2001). The different means by which astrocytes detect and remove synaptically-released glutamate are presented in section “Detection of Synaptically-released Glutamate by Astrocytes”.

Transport of glutamate across the astrocytic cell membranes coincides with transmembrane flux of sodium, potassium, protons and chloride and a decrease in the membrane potential (Marcaggi and Attwell, 2004; Rose et al., 2016). The tiny processes and compartments of astrocytes may be sensitive to small ion fluxes associated with the release of individual synaptic vesicles, and high frequency or prolonged activity of neurons could alter intracellular ion concentrations and consequently transport capacity substantially. Metabotropic glutamate receptors activated by synaptic activity have been shown to cause global or local changes in intracellular calcium concentration. Such calcium transients can result in astrocytic gliotransmitter release which feeds back to neurons (Perea et al., 2009; Araque et al., 2014). Thus, the spatial and temporal patterns of intracellular ion concentration changes are important parameters for glutamate-related effects induced in and by astrocytes. The section “Role of Astrocytic Ion Signals for Glutamate Homeostasis” discusses such activity-dependent intracellular ion concentration changes in response to binding and transport of glutamate.

Astrocytes display especially delicate cellular processes, which, in contrast to other cells in the brain, are far beyond the resolution achievable using classical diffraction-limited microscopy (Ventura and Harris, 1999). There are several structural factors that could facilitate uptake by these astrocytic processes: a large surface area, close spatial association with synapses and axons, and strong coupling to neighboring astrocytes. Not only is the communication with the ECS critically shaped by the sub-micron structure of astrocytes: The very narrow diameter of their fine tube- and sheet-like processes likely has important implications for intracellular ion distribution and may create multiple functionally and diffusibly almost independent compartments. As a consequence, the finest morphological changes, invisible to conventional light microscopy, could significantly modify the essential functions of astrocytes. The role of sub-micron scale astrocytic structural changes and their dynamics are discussed in section “Regulation of Synaptic Transmission by Astroglial Glutamate Transporters and Perisynaptic Astrocyte Structure” with particular reference to the strategic positioning of glutamate transporters to perform glutamate uptake.

## Tools to Investigate Astrocytic Responses to Glutamate

### Functional Analysis Using Electrophysiology and Optical Approaches

The impact of synaptically released glutamate on astrocytes and its functional consequences can be monitored by combining electrophysiology and pharmacological tools. While electrophysiological measurements represent a reliable and unambiguous method for functional detection of ion flux across the plasma membrane, it is worth keeping in mind that they are generally obtained from astrocyte somata and thus only provide a distant, filtered version of electrical signals generated in processes remote to the soma. This is especially critical when considering the low input resistance of astrocytes, resulting in a substantial loss of current with increasing distance from its site of generation, and in a poor control of the membrane potential in voltage-clamp experiments. In addition, astrocytes form electrically coupled networks via gap junctions, further complicating accurate measurement of individual cells (Giaume et al., 2010). Notwithstanding their very low input resistance, glutamate not only excites neurons but also depolarizes astrocytes as observed in early microelectrode recordings (Bowman and Kimelberg, 1984; Kettenmann et al., 1984).

In functional experiments, astrocytes may be identified by their typical morphology, by staining with sulforhodamine (SR 101; Nimmerjahn et al., 2004; Kafitz et al., 2008) or by the expression of fluorescent marker proteins (e.g., GFP) under the control of an astrocyte-specific promoter such as glial fibrillary acidic protein (GFAP; Zhuo et al., 1997). Application of glutamate or agonists of subtypes of ionotropic glutamate receptors (iGluRs) such as N-methyl-D-aspartate (NMDA), cis-1-amino-1,3-dicarboxycyclopentane (cis-ACPD), α-amino-3-hydroxy-5-methyl-4-isoxazolepropionic acid (AMPA) or kainate, in combination with specific antagonists, among them (R)-3C4HPG for NMDA receptors, 6,7-dinitroquinoxaline-2,3-dione (DNQX) for AMPA/kainate receptors or GYKI53655 for AMPA receptors, allowed a more detailed functional investigation of astrocytes’ electrophysiological responses to glutamate (Verkhratsky and Steinhäuser, 2000; Zhou and Kimelberg, 2001; Matthias et al., 2003; Zhou et al., 2006; Verkhratsky and Kirchhoff, 2007a). The specificity of such pharmacological manipulations, however, is not sufficient to further discriminate between the various iGluR subunit compositions, which could be addressed by using transgenic knockout animals.

**Figure 1:**
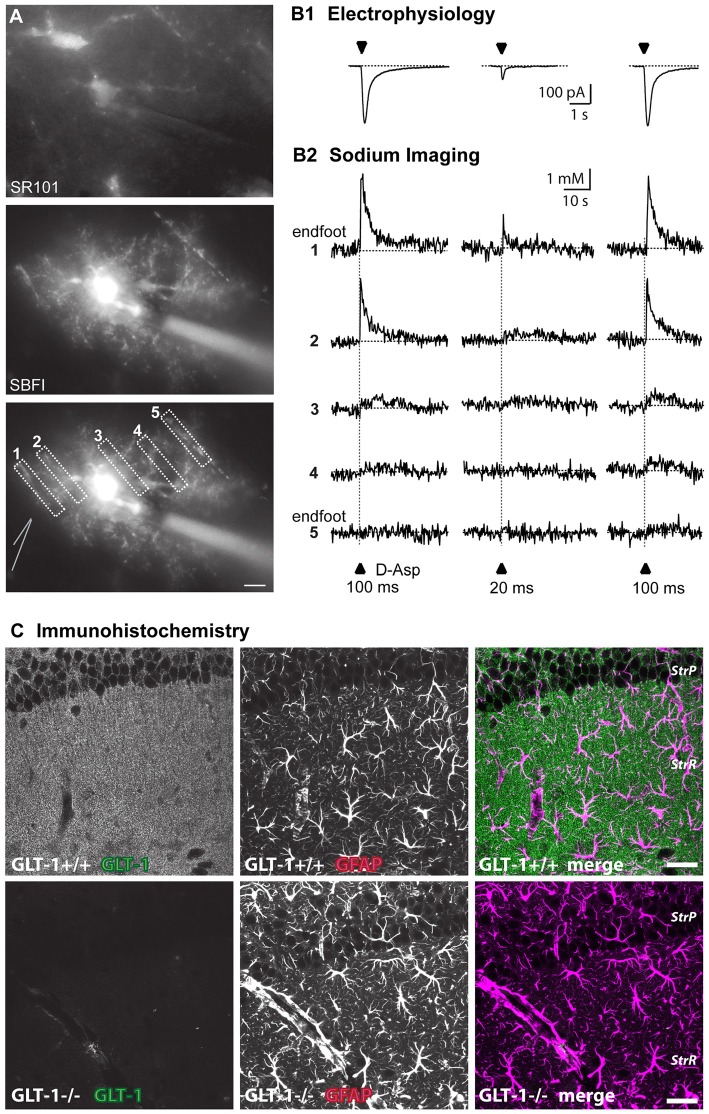
Approaches to visualize astroglial glutamate signaling and uptake. **(A)** Top: image of SR101-staining of astrocytes in the stratum radiatum. Center: epifluorescence image of an astrocyte selectively loaded with SBFI via a patch pipette (visible on the right side). The astrocyte contacts blood vessels both at the left and the right side. Bottom: same image as above with ROIs indicated from which measurements shown in **(B)** were taken. The position of the D-aspartate application pipette is schematically indicated at the left side. Scale bar: 10 μm **(B)** somatic inward currents elicited by the glutamate transporter agonist D-aspartate (arrowheads) for 100 ms or 20 ms as indicated **(B1)**. Shown below **(B2)** are sodium signals from the different ROIs as denoted in **(A)** that accompanied the inward currents. Note that signals are largest in ROI1 which covers an endfoot.** (C)** Immunohistochemical labeling of glutamate transporter 1 (GLT-1; EAAT2) in wild-type (upper row) and GLT-1 k.o.-animals (bottom row). Images represent maximum projections of five consecutive optical CLSM sections (1 μm) of the hippocampal CA1-region of P22. In addition to GLT-1 (left images), glial fibrillary acidic protein (GFAP) was labeled (middle panel), the merge is shown on the right. Scale bar: 20 μm. StrP, *stratum pyramidale*; StrR, *stratum radiatum*.** (A,B)** taken from Langer et al. (2017); **(C)** taken from Schreiner et al. (2014).

High-affinity glutamate transporters are electrogenic and can thus also be analyzed using electrophysiological techniques such as whole-cell patch-clamp (Figures 1A,B). Electrophysiological approaches to study glutamate transporter currents in astrocytes and to simultaneously record neuronal and astrocytic activity are summarized in recent reviews (Dallérac et al., 2013; Cheung et al., 2015). Indeed, the inward current generated by activation of glutamate transporters upon agonist application or electrical stimulation of afferent fibers is a reliable, semi-quantitative measure for the functional expression of glutamate uptake in glial cells (Brew and Attwell, 1987; Barbour et al., 1988; Bergles and Jahr, 1997; Bergles et al., 1997; Diamond and Jahr, 1997). Expression of glutamate transporters can be down-regulated by inhibition of their synthesis using chronic antisense oligonucleotide administration (Rothstein et al., 1996). Several pharmacological agents, such as ceftriaxone, estrogen, tamoxifen and riluzole increase the expression of glial glutamate transporters at the transcriptional level via activation of nuclear factor κB (Karki et al., 2015). Acutely, different transporter subtypes can be specifically blocked by substances like UCPH101 (2-amino-5,6,7,8-tetrahydro-4-(4-methoxyphenyl)-7-(naphthalen-1-yl)-5-oxo-4*H*-chromene-3-carbonitrile) or dihydrokainic acid (DHK), whereas DL-threo-beta-benzyloxyaspartate (DL-TBOA) and its high-affinity analog TFB-TBOA are non-specific blockers which can be used to inhibit all known transporter isoforms (Shimamoto et al., 1998; Abrahamsen et al., 2013; Tse et al., 2014).

In addition to electrophysiology, imaging of intracellular ion transients can be employed for detecting the activation of glutamate receptors as well as transporters in astrocytes. This is especially relevant for metabotropic receptors for glutamate (mGluRs). For analysis of the latter, imaging of related intracellular signaling components, such as calcium or cyclic adenosine monophosphate (cAMP), combined with specific pharmacological tools, is usually employed (Pasti et al., 1995; Niswender and Conn, 2010; see also section “Metabotropic Receptors”). The glutamate-induced calcium signals can be monitored by either synthetic or genetically encoded fluorescence-based reporter molecules. A detailed description of these ratiometric and single-wavelength sensors is outside the scope of this review (for recent surveys see Khakh and McCarthy, 2015; Rusakov, 2015). A general advantage of imaging based methods is their spatial resolution, which provides further information on the site of origin and the propagation of intra- and intercellular signals.

Imaging can also reveal activation of glutamate transport, which is driven by cotransport of different ions, among them sodium (Marcaggi and Attwell, 2004; Rose et al., 2016; Figure 1B). Depending on the strength of stimulation, sodium signals induced by glutamatergic activity in astrocytes can be local or global (Rose and Verkhratsky, 2016), thus allowing functional activation of glutamate transport in astrocyte microdomains such as perisynaptic processes or endfeet (Langer and Rose, 2009; Langer et al., 2017). Genetically encoded voltage sensors could also be powerful tools for imaging local depolarizations in astrocytes, but currently used voltage sensors lack the sensitivity to quantify small voltage changes (Yang and St-Pierre, 2016).

Another important technical challenge is the specific activation of astrocytes (Li et al., 2013). Purely pharmacological approaches, such as the application of receptor agonists, do not provide sufficient specificity for studies *in situ* or *in vivo*, and even pharmaco-genetic approaches, which have excellent cell-type specificity (Fiacco et al., 2007), do not mimic the precise temporal and spatial aspects of astrocyte activation. Electrically evoking neuronal neurotransmitter release, e.g., via stimulation of afferent fibers can be used as an indirect, but more physiological means for astrocyte activation.

Temporally and spatially defined activation of astrocytes can also be obtained by optical techniques, such as photo-uncaging of glutamate. For instance, local photo-uncaging of 4-methoxy-7-nitroindolinyl- (MNI-) caged glutamate proved to be useful for mapping the distribution of glutamate transporters on individual astrocytes (Armbruster et al., 2014). Exclusive specificity for astrocytes can be achieved with optogenetic approaches, i.e., by using genetically encoded, light-sensitive tools that can be targeted to the desired cell types and brain regions. One example is the expression of channelrhodopsin, a light-gated ion channel isolated from green algae, which can be stimulated with blue light to achieve depolarization and to induce the influx of calcium. Channelrhodopsin has been successfully used in a number of studies addressing the physiological function of astrocyte signaling *in vivo* (Gradinaru et al., 2009; Gourine et al., 2010; Sasaki et al., 2012; Perea et al., 2014). Another optogenetic approach, which might be useful for controlling astrocyte signaling, is the use of light-gated glutamate receptors. In this approach, photo-switchable ligands are employed to control specific iGluRs or mGluRs with high precision (Levitz et al., 2013; Reiner et al., 2015; Berlin et al., 2016), mimicking their physiological activation as closely as possible. This technique has also been used in cultured astrocytes, where activation of a light-gated kainate receptor enabled induction of calcium signals, demonstrating astrocyte-to-astrocyte signaling (Li et al., 2012).

### Immunocytochemistry to Study Protein Localization

During the last decades, confocal microscopy and multiphoton excitation microscopy, typically used for documentation of immunocytochemical and histochemical labeling, have been employed to directly visualize the distribution of glutamate receptors and transporters at the protein level. The utilization of antibodies against ionotropic and metabotropic glutamate receptors allowed analysis of their widespread distribution across neurons and glia (Martin et al., 1993; Petralia and Wenthold, 1998; Keifer and Carr, 2000; Aronica et al., 2001; Lee et al., 2010; Minbay et al., 2017). Today, a variety of antibodies directed against almost all known glutamate receptor subunits are available. The first antibodies against glutamate transporters became available in 1991 (Danbolt et al., 1992). Since that time, a substantial amount of knowledge has accumulated on the cellular localization and spatial and temporal distribution pattern of different subtypes of glutamate transporters (Rothstein et al., 1994; Furuta et al., 1997; Danbolt, 2001; Schreiner et al., 2014; Danbolt et al., 2016; Figure 1C).

Although these laser-scanning techniques are able to reveal not only laminar, but also cellular distribution patterns of receptor or transporter expression (see Figure 1C), they cannot provide information on the exact localization of antigens on fine astrocytic protrusions. The size of these structures (50–200 nm), which surround or approach excitatory synapses and are therefore often termed perisynaptic astrocytic processes (PAPs), is beyond resolution of conventional optical microscopy (200–300 nm; Derouiche and Frotscher, 1991; Witcher et al., 2007; Heller and Rusakov, 2015). The combination of optimized brain tissue clearing strategies like CLARITY (Chung et al., 2013) with super resolution confocal imaging using advanced Airy pattern reassignment strategies (first described by Sheppard (1988) and now implemented as Zeiss Airyscan (Huff, 2015)) improved the spatial resolution and enabled the counting and morphological reconstruction of astroglial processes in thick tissue (Chen et al., 2015; Miller and Rothstein, 2016).

In contrast to conventional light microscopy, electron microscopy (EM) provides sufficient resolution to identify and document the thinnest astroglial protrusions. Employing EM studies in combination with three-dimensional reconstruction techniques has allowed the identification of the fine structure of PAPs in various brain regions (Ventura and Harris, 1999; Grosche et al., 2002; Reichenbach et al., 2010; Heller and Rusakov, 2015). Pre- and post-embedding labeling of proteins of interest helped to further address the subcellular localization of glutamate receptors and transporters. The pre-immunoperoxidase labeling method, however, does not usually result in statistically reliable data and may not allow differentiation between proteins localized on the astrocytic membrane and neighboring neuronal membrane, since diffusion of the reaction product, diaminobenzidine, is a well-known technical limitation for precise localization (Ottersen and Landsend, 1997; Nusser, 2000). In contrast to this, post-embedding EM studies using the immunogold technique enables an estimation of various parameters e.g., the number, density and variability of receptors as well as transporters for glutamate at synapses (Chaudhry et al., 1995; He et al., 2000; Furness et al., 2008; Zhang et al., 2016).

### Super-resolution Microscopy and Protein Dynamics

EM with its superior spatial resolution, however, does not enable monitoring of live cells in real time, and thus provides only a static image of glutamate receptor and transporter distribution. The key to address this challenge lies in the utilization of super-resolution imaging techniques in live tissue that -under optimal conditions- overcome resolution limits of conventional light microscopy. Heller and Rusakov provide a detailed discussion of various super-resolution methods (i.e., stimulated-emission depletion (STED) microscopy, single-molecule localization imaging, such as photo-activated localization microscopy (PALM) or stochastic optical reconstruction microscopy (STORM), structured illumination microscopy (SIM)) relevant to study astrocytic glutamate transport (Heller and Rusakov, 2015). Stochastic super-resolution approaches like PALM/STORM build on the advantage that the specific labels emit light at separate time points and thereby become resolvable in time and thus diffraction limited imaging systems can be used.

Various approaches have been developed to follow membrane proteins through space and time (Kim et al., 2010; Rusakov et al., 2011). Single particle tracking microscopy employs a comparable strategy by tracking single structures rather than labeling a complete ensemble of proteins simultaneously (Saxton and Jacobson, 1997). This technique was successfully applied to study diffusion dynamics of neurotransmitter receptors and glutamate transporters, sparsely labeled with quantum dots, in subcellular domains of the astrocyte membrane (Arizono et al., 2012; Murphy-Royal et al., 2015; Al Awabdh et al., 2016). An alternative and less complex approach to study the kinetics of protein diffusion or transport is by fluorescence recovery after photobleaching (FRAP). For this approach, a protein label is bleached in a defined, restricted regions and the fluorescence recovery is monitored (Axelrod et al., 1976). Recovery times then yield information about the kinetics of protein diffusion or transport.

Diffusion of fluorescent proteins (FPs) can be further studied with fluorescence correlation spectroscopy (FCS; Magde et al., 1974), where the correlation in fluorescence fluctuation can be used to obtain both fluorophore concentration and diffusion characteristics. This allows accurate determination of the diffusion of plasma membrane proteins in defined compartments, for example spines, at diffraction-limited resolution. To date, however, only few studies have used FCS to monitor the diffusion properties of specific membrane proteins (Kim et al., 2010). One reason may be that high-end instrumentation with excellent signal-to-noise ratio and low concentrations of highly fluorescent fluorophores are required for successful implementation of FCS. By combining the techniques of super-resolution microscopy, single molecule imaging and electrophysiological recordings, knowledge on the surface dynamics of neurotransmitter receptors, transporters and ion channels in astrocytes could be substantially expanded (Ciappelloni et al., 2017).

### Monitoring Glutamate Levels and Dynamics

Recently, several concepts were developed to directly monitor glutamate levels both inside and at the surface of living cells. The periplasmic glutamate/aspartate-binding protein GltI from *Escherichia coli* provides an attractive scaffold, and forms the basis on which various sensors were constructed. The ligand-dependent conformational change in GltI has been used to create glutamate sensors from small molecule dyes coupled to single introduced cysteines (de Lorimier et al., 2002). Similarly, EOS (for E (glutamate) optical sensor (OS)), is a fluorescent sensor, which is based on the glutamate-binding domain of the AMPA receptor subunit GluR2 and a small fluorescent molecule conjugated near the glutamate-binding pocket. Thus EOS changes its fluorescence intensity (by maximally 37%) upon binding of glutamate, for which it has both high affinity (dissociation constant of 148 nM) and high selectivity (Namiki et al., 2007). With improved EOS variants, it was possible to detect extrasynaptic glutamate activities in acute slice preparations (Okubo et al., 2010). It must be noted that these kinds of sensors require chemical synthesis and cannot be endogenously expressed by the cell system.

GltI was further functionalized as an *in vitro* glutamate sensor, and was thus employed as a recognition element for developing a fluorescent indicator protein for glutamate (FLIPE; Okumoto et al., 2005). This biosensor is based on Förster resonance energy transfer (FRET), the radiationless energy transfer from a donor to acceptor fluorophore where its efficiency depends on distance, orientation and spectral properties of the fluorophores (Förster, 1948). In FLIPE the hinge-bending motion upon glutamate binding leads to a conformational change which is transduced into a glutamate-dependent change in FRET efficiency between the attached enhanced cyan fluorescent protein (CFP) and Venus, acting as donor and acceptor, respectively. The resulting acceptor/donor emission ratio change is inversely correlated to the change in glutamate concentration. The FLIPE sensor detects glutamate but also aspartate with 10-fold lower affinity and glutamine with 100-fold lower affinity. By creating FLIPE variants with different affinities, it is suitable for applications such as visualizing glial glutamate metabolism, transport and spill-over effects (Okumoto et al., 2005).

At the same time, Hires et al. (2008) created glutamate-sensitive fluorescent reporters (GluSnFRs) by using FRET between cyan and yellow fluorescent proteins bracketing the GltI protein (Tsien, 2005). This variant was later improved by systematic optimization of linker sequences and glutamate affinities and the resulting SuperGluSnFR exhibits a 6.2-fold increase in response magnitude over the original GluSnFR. Quantitative optical measurements revealed the time course of synaptic glutamate release, spill-over, and reuptake with sub second temporal and spine-sized spatial resolution (Hires et al., 2008). So far, however, these sensors were rarely used beyond initial proof-of-principle experiments. Moreover, the authors of the latter paper discussed the need to develop sensors that can be targeted to specific subcellular regions, e.g., the active zone, to enable a distinction between synaptic and extrasynaptic changes in glutamate.

Ratiometric FRET sensors provide several advantages and drawbacks compared to single-wavelength indicators. In theory, ratiometric sensors facilitate quantification of the ambient ligand concentration because the ratio of fluorescence intensities should be independent of sensor concentration and concentration changes. However, FRET sensors often have a low dynamic range and therefore display small ratio changes upon ligand binding. Also, ratiometric measurements consume greater spectral bandwidth and may require multiplexing (Piston and Kremers, 2007). Single-wavelength indicators, typically based on circularly permuted or split FPs, although not being ratiometric, are an appealing alternative to FRET sensors. iGluSnFR provides a strong fluorescence change upon glutamate binding with an affinity of about 5 μM when expressed on a neuronal membrane (although varying considerably depending the exact expression system and environment) and has been used in different experimental settings. For instance, iGluSnFR enabled to monitor neuronal glutamate release at single spines (Marvin et al., 2013) and from single synapses (Jensen et al., 2017) and to reveal that glutamate released from mossy fibers reaches astrocytes in micromolar concentrations (Haustein et al., 2014). It has also been applied in *in vivo* experiments (Marvin et al., 2013; Hefendehl et al., 2016) and to investigate the mechanisms than control cortical glutamate uptake *in situ* (Armbruster et al., 2016). These recent experiments using iGluSnFR highlight its versatility.

## Detection of Synaptically-Released Glutamate by Astrocytes

Astrocytes detect synaptically-released glutamate by its binding to ionotropic and metabotropic glutamate receptors. Furthermore, astrocytes express high-affinity glutamate transporters, which represent the most important mechanism for removal of glutamate from the ECS. Both mechanisms result in the generation of intracellular signals, either by direct ion transport across the plasma membrane or by induction of store-mediated calcium release and/or other second messengers as discussed below.

### Astrocyte Glutamate Receptors

#### Ionotropic Receptors

Expression of ionotropic glutamate receptors by astrocytes is astonishingly heterogeneous and differs between brain regions (Verkhratsky and Kirchhoff, 2007b; Verkhratsky, 2010). There is clear evidence for functional expression of AMPA receptors on Bergmann glial cells of the cerebellar cortex, which are activated by ectopic release of glutamate at parallel fiber as well as climbing fiber synapses (Matsui et al., 2005). These have a relatively high calcium permeability and calcium signals resulting from AMPA receptor opening have been shown to control proper coverage of Purkinje cell synapses by Bergmann glia appendages (Iino et al., 2001). Retraction of Bergmann processes following deletion of AMPA receptors or their conversion to calcium-impermeable forms slowed the decay of excitatory postsynaptic potentials (EPSPs) in Purkinje cells by delaying the removal of glutamate at the synapse, resulting in an impairment of fine motor coordination (Iino et al., 2001; Saab et al., 2012). Therefore, it appears that AMPA receptors on Bergmann glial cells mediate an intricate interplay between glial calcium signaling, close ensheathment of synapses by perisynaptic glial processes and clearance of glutamate in the cerebellar cortex.

In the forebrain, AMPA receptor expression by astrocytes has been described in the neocortex (Lalo et al., 2006; Hadzic et al., 2017). They only mediate a small portion of the inward current induced by synaptic release of glutamate (the majority being carried by electrogenic glutamate uptake) and their functional relevance is as of yet unclear (Lalo et al., 2011). Moreover, there is clear evidence for AMPA receptors on processes, but not somata of radial-like glial cells in the subventricular zone of the dentate gyrus (Renzel et al., 2013). Astrocytes in the hippocampus apparently lack AMPA receptors, as opposed to NG2 cells, which were previously often classified as “immature”, “complex” or “rectifying” astrocytes (Jabs et al., 1994; Latour et al., 2001; Zhou and Kimelberg, 2001; Matthias et al., 2003). A similar picture emerges for NMDA receptors. These have been identified on the mRNA, protein, and functional level in cortical astrocytes (Conti et al., 1996; Schipke et al., 2001; Lalo et al., 2006). While there are clear indications for the involvement of NMDA receptors in astroglial signaling in hippocampal astrocytes (Porter and McCarthy, 1995; Serrano et al., 2008; Letellier et al., 2016), there is still no unequivocal evidence for their expression in these cells, and their presence therefore remains subject to discussion (Matthias et al., 2003; Verkhratsky and Kirchhoff, 2007b; Lalo et al., 2011; Dzamba et al., 2013).

**Figure 2:**
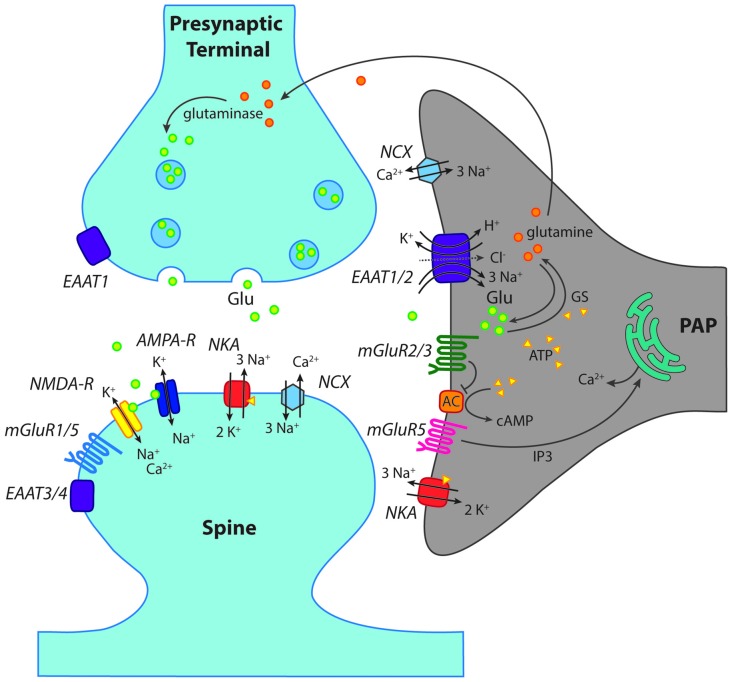
Scheme of a “tripartite” glutamatergic synapse in the hippocampus. Shown is a presynaptic terminal and a postsynaptic spine as well as a perisynaptic astrocyte process (PAP) reaching close to the synaptic cleft. In addition to ionotropic (NMDA, AMPA) and metabotropic (mGluR) receptors for glutamate, glutamate transporters (EAAT1–4) are indicated. Moreover, main mechanisms of ion transport across the plasma membrane, such as the Na^+^/K^+^-ATPase (NKA) are schematically shown. GS, glutamine synthase; AC, adenylate cyclase; NCX, Na^+^/Ca^2+^-exchanger. For further explanations and abbreviations: see text.

Suffice it to say, in contrast to Bergmann glial cells or NG2 cells, there is as yet no indication that AMPA or NMDA receptors might play any functional role in astrocytes in the hippocampus. Due to this, they will no longer be discussed in this review (see Figure 2). Why there is such heterogeneity in the expression profile of ionotropic glutamate receptors on different types of macroglial cells is unknown.

#### Metabotropic Receptors

In contrast to iGluRs, expression of metabotropic glutamate receptors (mGluRs) has been firmly established for astrocytes in the hippocampus (Schools and Kimelberg, 1999; Tamaru et al., 2001; Aronica et al., 2003). The most prominent role has long been attributed to mGluR5, a member of the group I mGluRs, which activates G_q_ and phospholipase C and has received particular attention as this results in the generation of calcium signaling by IP_3_-mediated release from intracellular stores (Figure 2; Pasti et al., 1995; Porter and McCarthy, 1996; Latour et al., 2001; Zur Nieden and Deitmer, 2006; Panatier and Robitaille, 2016).

While this concept apparently holds true for the juvenile hippocampus, it was recently called into question for the adult brain, where application of mGluR5 agonists failed to induce calcium signaling in astrocyte somata (Sun et al., 2013). Moreover, in the mature hippocampal mossy fiber pathway, it was found that mGluR5 was only partially responsible for calcium signals induced by axonal glutamate release in astrocyte processes (Haustein et al., 2014). While differences may be related to different variations in experimental approaches (Panatier and Robitaille, 2016), these results clearly demonstrate that calcium signaling in astrocytes is still not completely understood. Employment of more advanced calcium imaging techniques like rapid 3D-scanning of astrocytes *in situ* and *in vivo* imaging approaches are needed to resolve this issue (Bazargani and Attwell, 2016; Shigetomi et al., 2016; Bindocci et al., 2017).

In addition, astrocytes express mGluR2/3, the activation of which is coupled to G_i/o_ and results in inhibition of the adenylate cyclase (AC; Figure 2; Schools and Kimelberg, 1999; Tamaru et al., 2001; Aronica et al., 2003). This signaling pathway causes suppression of cAMP levels, and is generally not regarded as being directly involved in astrocyte calcium signaling (Sun et al., 2013). Notwithstanding this, activation of mGluR2/3 has recently been related to the generation of slow calcium transients in astrocytes in the mossy fiber pathway (Haustein et al., 2014). The mGluR2/3-induced calcium signaling was attributed to the action of G-protein β/γ subunits activating phospholipase C and interaction with IP_3_ receptors (Haustein et al., 2014), in analogy to GABA_B_ receptors.

### High-affinity Glutamate Transporters

Although receptors allow cells to react to synaptically-released glutamate, arguably the biggest impact on extracellular glutamate itself is made by high-affinity glutamate transporters, the EAATs. Not only do these prevent excitotoxicity through the removal of glutamate from the cleft and the ECS, but in doing so they also act as a vital component of plasticity and synaptic function. A total of five different mammalian subtypes of EAAT have been identified (Danbolt, 2001), and these show distinctive expression patterns throughout the cortex (Arriza et al., 1994). The different isoforms all adhere to the same general glutamate uptake stoichiometry; importing one glutamate molecule into the astrocyte by using the energy gained from co-transporting three sodium ions and one proton down the electrochemical gradients, whilst also exporting one potassium ion (Figure 2; Nicholls and Attwell, 1990). While all of these subtypes thus couple cations to glutamate transport in the same ratios, they also exhibit and uncoupled anion conductance, and therefore act as chloride ion channels (Amara and Fontana, 2002; Fahlke and Nilius, 2016). Isoforms are functionally distinguished from each other via glutamate transport rates, substrate affinities and accompanying chloride conductance (Arriza et al., 1994; Maragakis and Rothstein, 2004; Rose et al., 2016).

The two predominant transporter isoforms present in the hippocampus are EAAT1 (rodent analog: GLAST; glutamate/aspartate-transporter) and EAAT2 (GLT-1; glutamate transporter 1). While various *K*_m_ (Michaelis-Menten constant, reflecting concentration of half-maximal occupancy) and *V*_max_ (maximal transport rate) values have been reported for these transporter species depending on the experimental system used, it is generally agreed on that EAAT2 is the more effective transporter with a higher turn-over rate than EAAT1 (Arriza et al., 1994). Additionally, EAAT3 (also known as EAAC1) can be found at post-synaptic neuronal membranes, albeit at a much lower density than astrocytic EAAT1 and 2 (Holmseth et al., 2012). Moreover, in the adult hippocampus, 10% of EAAT2 transporters are also expressed on neuronal axon terminals (Danbolt, 2001).

Transporter function is critical throughout brain development, and double knockout animals of EAAT 1 and 2 are not viable and display cortical abnormalities and disorganization (Matsugami et al., 2006). General ablation of only EAAT2 has severe effects as well and animals die soon after birth from major seizures (Rothstein et al., 1996; Tanaka et al., 1997). Importantly, knocking out EAAT2 specifically in astrocytes mimics these effects, demonstrating the vital relevance of glial glutamate uptake for glutamate homeostasis in the brain (Petr et al., 2015). Consistent with this view, knockout animals for the other transporters, including EAAT1, show only weak to moderate impairment (Watase et al., 1998).

In the neonatal hippocampus, astrocytes are still immature and developing their processes that will later define their distinct cellular domains (Bushong et al., 2004). The astrocytes in this phase mainly rely on the subtype EAAT1 for glutamate transport and overall transport levels have been shown to be lower than in mature astrocytes (Diamond, 2005). Expression of this subtype increases over the first two postnatal weeks and then remains stable (Ullensvang et al., 1997; Schreiner et al., 2014). EAAT2 later takes over the bulk of hippocampal glutamate transport capacity, accounting for more than 90% of total uptake (Zhou et al., 2014). Its expression is delayed by around 10 days, starting to appear at p10–15 and increasing as cells mature until plateauing at p20–25 (Ullensvang et al., 1997; Schreiner et al., 2014). While this change in expression pattern has been shown to be regulated by neuronal activity (Swanson et al., 1997), the reason for the switch is unclear, especially as it is not universal across brain regions. For example, the same change happens in the cortex, but is delayed by around 2 weeks (Hanson et al., 2015), while Bergmann glia continue to primarily express EAAT1 into adulthood (Rothstein et al., 1994).

### Influence of Glutamate Transporters on Extracellular Glutamate Homeostasis

Extracellular glutamate has a diffusion coefficient of ~0.3–0.5 μm^2^/ms (Zheng et al., 2008). While this suggests that glutamate will rapidly leave the synaptic cleft by diffusion after activity, it remains low at rest outside of the cleft, with a concentration of around 20–60 nM (Selkirk et al., 2005; Herman and Jahr, 2007; Yamashita et al., 2009). This is because astrocytes are very effective at clearing glutamate after activity. Indeed, in the rat hippocampus, glutamate transporters are not overwhelmed even during high frequency stimulation (Diamond and Jahr, 2000). However, in the mouse cortex, a considerable slowing of glutamate uptake following bursts of presynaptic action potentials was observed (Armbruster et al., 2016). The transport cycle, which exhibits turnover rates of 16 s^−1^ for EAAT1 (Wadiche and Kavanaugh, 1998) and ~10–40 s^−1^ for EAAT2 (Wadiche et al., 1995; Bergles and Jahr, 1997; also see Danbolt, 2001), is relatively slow. This has lead to the idea that a major role of glutamate transporters is the fast buffering of synaptically released glutamate (Wadiche et al., 1995; Diamond and Jahr, 1997; Danbolt, 2001; Diamond, 2005). Given the high glutamate buffering and uptake capacity of transporters, the question arises how astroglial release of the gliotransmitter glutamate is coordinated with glutamate uptake, in other words if and how the immediate buffering/uptake of glutamate released by astrocytes is prevented. One potential scenario is the spatial segregation of astroglial glutamate uptake and glutamate release, which to our knowledge is not supported by any direct experimental data at the moment.

Extracellular glutamate concentrations outside of the synaptic cleft have been calculated to exceed 1 μM when 95% of astrocytes are no longer functional (Zheng et al., 2008). Failure to this extent can have critical consequences as it reduces signal-to-noise ratios at synapses and allows transmitter spill-over, which results in synaptic crosstalk (Asztely et al., 1997; Trabelsi et al., 2017). This effect can be mimicked through the deletion of astrocytic EAAT2, which produces epileptic activity (Petr et al., 2015) and subsequent excitotoxicity (Selkirk et al., 2005). Although neurons have some mechanisms to help them cope with higher glutamate levels, e.g., through the regulation of Kv2.1 channel clustering and phosphorylation after perisynaptic NMDA receptor activation (Mulholland et al., 2008), extended periods of elevated extracellular glutamate do cause irreversible damage to the surrounding cells.

While the most important determinant for sustained activation of NMDA receptors has been suggested to be release of glutamate upon presynaptic discharges (Zheng and Rusakov, 2015), transporters also have to be constantly active to counteract accumulation of tonically released glutamate (Jabaudon et al., 1999). This tonic release contributes to a constant stimulation of extrasynaptic NMDA receptors, which remains unaltered by additional saturation with glycine, therefore indicating that glutamate is the limiting factor of this activation (Le Meur et al., 2007). The glutamate release responsible for this was originally attributed to the action of the cysteine-glutamate exchanger, but it was later found that the blocking of its action hardly altered the resulting NMDA receptor activation (Cavelier and Attwell, 2005). It has now been shown that this tonic activation can be significantly reduced by inhibiting the glutamate-glutamine cycle in astrocytes, thereby identifying them as the primary source of the ambient synaptic glutamate levels (Cavelier and Attwell, 2005; Le Meur et al., 2007).

This glutamate-glutamine cycle is a critical component of hippocampal astrocyte function, wherein glutamate taken up by transporters is converted into non-toxic glutamine by glutamine synthase (GS) and ATP, before being transported back into neurons (Figure 2). Pharmacological inhibition of GS results in significantly larger and longer NMDA receptor currents in cortical pyramidal neurons upon high-frequency stimulation, indicating that suppression of enzyme activity elevates intracellular glutamate levels and impairs glutamate uptake (Trabelsi et al., 2017). Neurons recycle glutamine back into glutamate or GABA in order to replenish transmitter stores (Schousboe et al., 2014). This is an essential process as neurons are unable to produce these transmitters *de novo* (Bak et al., 2006). Interestingly, astrocytes also contain the enzyme glutaminase required to produce glutamate from glutamine (Cardona et al., 2015). Although the cycle has been proven critical to the glial release of transmitter, the exact release mechanisms remain unclear. However, this “gliotransmission” occurs independently of calcium, and it has been suggested that it may happen via a VAMP3 mechanism, regulated by cAMP (Li et al., 2015). Although the function of this release and uptake cycle remains unresolved at present, astrocytes seem to be the main mediator of both processes, which are important for the rapid turn-over rate of extracellular glutamate within the hippocampus (Jabaudon et al., 1999).

## Role of Astrocytic Ion Signals for Glutamate Homeostasis

As mentioned above, activation of astrocyte glutamate receptors as well as glutamate uptake through high-affinity transport leads to changes in intracellular ion concentrations. Such ion concentration changes, in turn, may act as signals to trigger release of glutamate from astrocytes and/or modulate glutamate transport activity and thereby represent relevant mediators of astrocytes’ control of extracellular glutamate homeostasis.

### Ion Signaling Related to Glutamate Transport

Astrocytes experience major changes of their cytosolic ion concentrations in response to glutamate because of the activity of high-affinity and sodium-dependent glutamate uptake. These ions include primarily sodium and protons (Rose and Ransom, 1996; Kirischuk et al., 2016), which are transported together with glutamate. Moreover, depending on the EAAT isoform expressed, glutamate transporters mediate a detectable flux of chloride (Untiet et al., 2017). The resulting degradation of ion gradients reduces its driving force and exerts a negative feedback on transport capacity, which may in extreme cases, e.g., during additional disturbance in ion homeostasis, even result in reversal of glutamate uptake. Ion signaling related to glutamate transport thereby represents an important modulator of astroglial control of extracellular glutamate.

Under physiological conditions, glutamate transporters exhibit a reversal potential in the far positive range (>50 mV, Barbour et al., 1991; Bergles and Jahr, 1997). Changes in the plasma membrane gradients of the transported ions (Na^+^, H^+^, K^+^) directly influence glutamate transport activity, and because of its transport stoichiometry, this is especially relevant for sodium ions (Szatkowski et al., 1990; Barbour et al., 1991; Zerangue and Kavanaugh, 1996; Levy et al., 1998). In addition, as glutamate uptake is electrogenic, the depolarization mediated by glutamate uptake, together with the depolarizing effect of activity-related increases in extracellular K^+^, will cause a dampening of further transport activity (Barbour et al., 1988; Szatkowski et al., 1990).

During periods of metabolic inhibition, reverse operation of glutamate transport can serve as a source for glutamate and contribute to its accumulation in the ECS as described for neurons under severe ischemia (Rossi et al., 2000). The same is true for reverse glutamate uptake by glia, which in isolated Müller glial cells has been shown to be induced by (very) high external potassium (Szatkowski et al., 1990). It is important to emphasize, however, that as compared to uptake of other transmitters such as GABA, glutamate uptake is highly robust. Reverse glutamate uptake in brain tissue as a mechanism for glia-mediated release of glutamate is often proposed but only possible with excessive cellular sodium loading together with strong depolarization, conditions usually only found upon complete metabolic inhibition and failure of Na^+^/K^+^-ATPase activity in ischemic core regions (see Rossi et al., 2000; Gerkau et al., 2017).

While reverse glutamate uptake may occur only under severe ischemic conditions, less dramatic changes in astrocyte ion gradients may still directly feed back onto extracellular glutamate levels by reducing the driving force for transport. The relevance of ongoing glutamate uptake is evident from the fact that its pharmacological inhibition causes an immediate increase in extracellular glutamate accumulation, accompanied by activation of neuronal glutamate receptors (Rothstein et al., 1996; Jabaudon et al., 1999) and rapid, fatal sodium loading of both neurons and astrocytes (Langer and Rose, 2009; Karus et al., 2015). Moderately increasing the cytosolic sodium concentration in astrocytes (to ~35–40 mM) is directly linked to a reduction of glial glutamate transport activity as demonstrated for astrocytes in hippocampal tissue slices exposed to elevated NH_4_^+^/NH_3_ concentrations (Kelly et al., 2009). The same is true for a moderate depolarization (by ~8–10 mV) of hippocampal astrocytes *in situ*, which—as expected based on the transport stoichiometry—significantly reduced the amplitude of glutamate uptake currents induced by application of the transporter agonist D-aspartate (Stephan et al., 2012).

These studies clearly show that if intracellular sodium rises in astrocytes, the reversal potential for glutamate transport is shifted in a negative direction, thereby reducing their glutamate uptake capacity. Notably, glutamate uptake simultaneously represents the most powerful pathway for the induction of sodium signals as demonstrated for astrocytes in the *stratum radiatum* of the hippocampal CA1 area (see Figure 1B; Langer and Rose, 2009; Karus et al., 2015; Langer et al., 2017), cerebellum (Kirischuk et al., 2007; Bennay et al., 2008), neocortex (Lamy and Chatton, 2011; Unichenko et al., 2013), and at the Calyx of Held (Uwechue et al., 2012). Activity-related sodium signals following activation of glutamate uptake are detectable in perisynaptic astrocyte processes, and their amplitudes have been shown to be related to the strength of synaptic stimulation over a wide range of stimulation intensities, reaching ~6 mM with 10 pulses, (Langer and Rose, 2009). If such sodium elevations, and the accompanying reduction in transport capacity, result in a reduction of overall glutamate uptake by astrocytes and thereby in a modulation of extracellular glutamate concentrations and synaptic glutamate transients under physiological conditions, remains to be established.

Besides the direct negative feedback effect of sodium elevations on glutamate transport capacity, there is also accumulating evidence that sodium signals in response to transport activation might drive reversal of the sodium-calcium exchange (NCX) in astrocytes (Figure 2; Kirischuk et al., 2012; Boscia et al., 2016). The consequence of such a reversal is the generation of calcium influx into astrocytes (Kirischuk et al., 1997; Song et al., 2013; Gerkau et al., 2017). Calcium signals induced by sodium-driven NCX might then result in calcium-dependent release of glutamate, again linking astrocyte sodium signals to their regulation and modulation of extracellular glutamate concentrations.

### Astrocyte Calcium Signaling

Astrocytes also detect synaptically-released glutamate by activation of glutamate receptors as described above. Their activation often leads to the generation of intracellular ion signals and/or second messengers (Figure 2). Transient astrocyte calcium elevations in response to glutamatergic activity were one of the first intracellular signals implicated in neuron-glia interaction at synapses (Enkvist et al., 1989; Cornell-Bell et al., 1990; Kim et al., 1994; Hassinger et al., 1995). While initial experiments were performed in cell culture, it soon became clear that also astrocytes *in situ* also respond to neuronal release of transmitters with increases in calcium, as for example shown in acute hippocampal tissue slices (Dani et al., 1992; Porter and McCarthy, 1996; Pasti et al., 1997). Later on, this concept was shown to hold true within the intact brain by demonstrating that hippocampal astrocytes *in vivo* undergo calcium signaling in response to neuronal activity (Kuga et al., 2011; Takata et al., 2011; Navarrete et al., 2012). The exact astroglial mechanisms generating calcium transients and their spatial and temporal properties are still under debate despite the wealth of experimental evidence (Agulhon et al., 2010; Volterra et al., 2014; Bazargani and Attwell, 2016; Rungta et al., 2016; Shigetomi et al., 2016; Bindocci et al., 2017).

An important cellular response to these calcium transients includes calcium-dependent release of neurotransmitters from astrocytes targeting nearby neurons and their synapses. The considerable experimental evidence for the existence of such feedback signaling, the involved mechanisms, the current controversies and open questions have been discussed in detail recently by ourselves and others (Hamilton and Attwell, 2010; Araque et al., 2014; Rusakov et al., 2014; Verkhratsky et al., 2016; Bohmbach et al., 2017). The relevance of astroglial calcium signaling for neurotransmitter uptake is less explored. As discussed above, calcium and sodium signaling are linked via the NCX such that sodium elevations may trigger calcium-entry via the NCX. Whether the reverse, sodium-entry as a consequence of calcium export through NCX, can modify the driving force of astroglial glutamate uptake (and thus its efficiency) significantly, has not been explored to the best of our knowledge.

In addition, calcium increases could regulate insertion and internalization of glutamate transporters. Such a direct effect of astroglial calcium levels on neurotransmitter transport has been demonstrated for the GABA transporters GAT3 in the hippocampus. Chelation of intracellular calcium by infusing astroglia with the calcium buffer BAPTA via a patch pipette reduced the GAT3 levels and induced a tonic, GABA-receptor-mediated current recorded in interneurons, indicating that lowering astroglial calcium levels reduces GAT3-mediated GABA uptake (Shigetomi et al., 2011). Direct evidence for the calcium-dependence of glutamate transport in astrocytes *in situ* is currently not available but several experimental observations make the existence of such a mechanism plausible. First, an increase of glutamate uptake by cultured astrocytes can be induced by basic fibroblast growth factor on a time scale of hours via a partially calcium-dependent signal cascade (Suzuki et al., 2001). Second, the down-regulation of astroglial glutamate uptake currents in spinal cord slices by interleukin 1beta within minutes of its application is likely to involve astroglial calcium signaling (Yan et al., 2014). Finally, the manipulation of calcium levels in cultured astroglia affected the insertion and removal of eGFP-tagged GLT-1 into and out of the membrane (Stenovec et al., 2008). Together these studies demonstrate that changes of astroglial calcium levels could directly control glutamate uptake. Because astrocyte calcium signaling itself depends on neuronal activity (see above), glutamate uptake could be finely tuned by synaptic activity via astroglial calcium signaling.

## Regulation of Synaptic Transmission by Astroglial Glutamate Transporters and Perisynaptic Astrocyte Structure

### Astroglial Transporters Constrain the Spread of Synaptically Released Glutamate

As discussed above, astrocytes take up the vast majority of synaptically released glutamate (>90%). However, glutamate is released from the presynaptic terminal directly into the synaptic cleft, which is typically devoid of astrocyte processes and thus astroglial glutamate transporters. It is therefore thought that astrocytes do not control the initial spread of synaptically released glutamate inside the synaptic cleft under physiological conditions (see below) and that instead diffusion and dilution of glutamate primarily underlies the dissipation of the steep glutamate concentration gradients directly after release (Clements et al., 1992; Danbolt, 2001; Scimemi and Beato, 2009). At these nanometer and microsecond scales, intra-cleft glutamate concentration transients have escaped direct observation. Instead, numerical simulations have been employed to analyze the intra-cleft spread of glutamate and its escape into the perisynaptic space that contains perisynaptic astrocyte processes (Diamond, 2001; Zheng et al., 2008; Scimemi and Beato, 2009; Allam et al., 2012; see section “Influence of Glutamate Transporters on Extracellular Glutamate Homeostasis” for glutamate binding to astroglial transporters and uptake).

**Figure 3:**
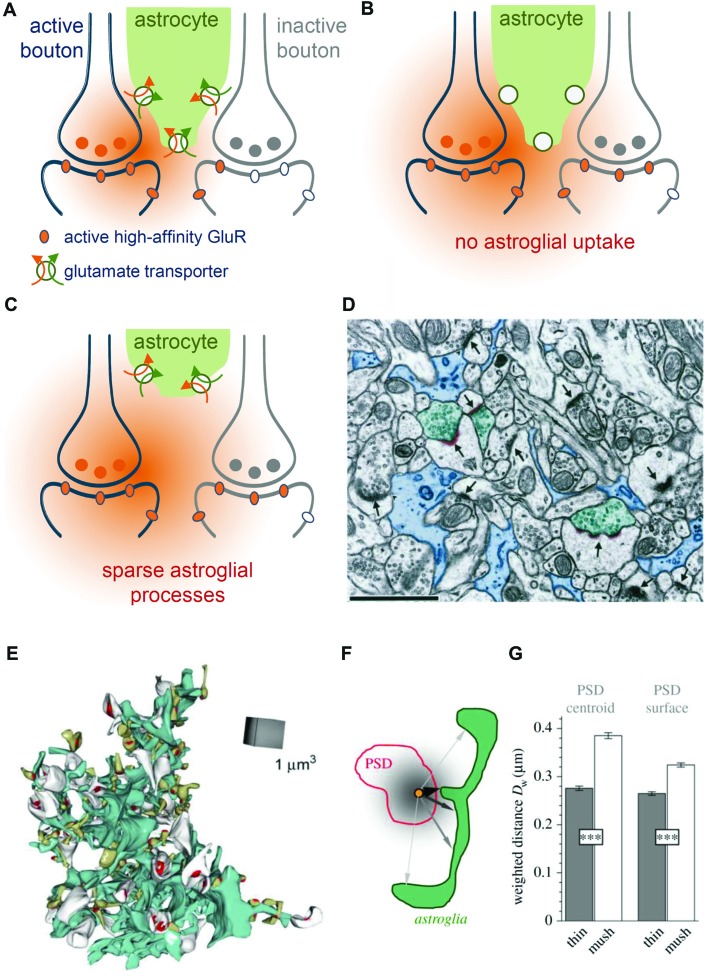
Role of astroglial transporters and perisynaptic astroglial morphology. **(A)** Synaptically-released glutamate (orange) diffuses in extracellular space (ECS) before astroglial (green) clearance (orange arrow) along with uptake of sodium takes place (green arrow). Released glutamate will activate high-affinity glutamate receptors (orange oval) at active synapses (blue, left) but also some on neighboring, presynaptically-inactive synapses (gray, right, gray ovals represent inactive glutamate receptors), leading to “glutamate spill-over” or synaptic crosstalk. **(B)** When glutamate uptake is pharmacologically inhibited, glutamate spreads further into extrasynaptic space and is more likely to activate high-affinity glutamate receptors at nearby synapses. **(C)** Perisynaptic astroglial process structure is expected to affect the local efficiency of astroglial glutamate clearance. For example, an increased distance of astroglial processes from an active synapse is likely to reduce the efficiency of local clearance/uptake and to increase the probability of synaptic crosstalk via high affinity glutamate receptors. **(D)** Not all synapses are directly contacted by perisynaptic astroglial processes. Adopted with permission from Ventura and Harris (1999). Astroglial processes (blue, scale bar 1 μm) were visualized using electron microscopy (EM) and their spatial relationship to synapses (arrows) was studied. Note, that only three synapses (arrowheads) are directly contacted by astroglial processes (see Ventura and Harris, 1999 for quantification) suggesting a highly uneven coverage of synapses by astroglial glutamate transporters. **(E)** Example of an EM 3D-reconstruction of a single astroglial fragment (turquoise) with adjacent postsynaptic spines (white and greenish) with their postsynaptic densities (PSD) from the dentate gyrus (Medvedev et al., 2014). **(F)** The local abundance of astroglial processes around individual spines was determined by the average, diffusion-weighted distance from the PSD center or surface to perisynaptic astroglia. Analysis revealed (see Medvedev et al., 2014 for more details) that astroglial processes were on average closer to thin than to mushroom spines **(G)** suggesting that perisynaptic glutamate transients at thin spines are more tightly controlled by astroglial transporters.

These simulations predicted that perisynaptic astrocyte branches and the glutamate transporters located on them reduce the probability of glutamate escaping into perisynaptic ECS and activating perisynaptic high-affinity receptors such as mGluRs or NMDA receptors (Zheng et al., 2008). Indeed, glutamate released at one synapse can activate high-affinity NMDA receptors at nearby synapses (a phenomenon called glutamate spill-over or synaptic crosstalk) and pharmacological blockade of glutamate transporters significantly exacerbates this process (Asztely et al., 1997; Diamond, 2001; Arnth-Jensen et al., 2002; Scimemi et al., 2004). Thus, astroglial glutamate transporters constrain but do not prevent synaptic crosstalk between hippocampal synapses via high-affinity NMDA receptors (Figures 3A,B).

Because astroglial glutamate transporters limit activation of NMDA receptors, it is likely that they also affect NMDA receptor-dependent synaptic plasticity. Indeed, the amplitude ratio of EPSCs mediated by NMDA and low-affinity AMPA receptors was increased in recordings from CA1 pyramidal cells in acute slices from GLT-1 knockout animals (Katagiri et al., 2001), suggesting a larger contribution of NMDA receptors. In the same preparation, long-term potentiation of CA3-CA1 synaptic transmission was impaired, which was attributed to excessive activation of NMDA receptors (Katagiri et al., 2001). In addition, up-regulation of GLT-1 expression by ceftriaxone impaired long-term depression and reduced long-term potentiation at mossy fiber-CA3 synapses (Omrani et al., 2009). A role of glutamate transporters for NMDA receptor-dependent synaptic plasticity has also been established outside of the hippocampus (Massey et al., 2004; Tsvetkov et al., 2004; Valtcheva and Venance, 2016), which indicates that the control of NMDA receptor-activation—and therefore NMDA receptor-dependent synaptic plasticity—by astroglial glutamate could be a ubiquitous phenomenon in the brain.

Glutamate transporters also control the activation of other high affinity glutamate receptors. For instance, mGluR activation of interneurons in response to synaptic glutamate release was strongly increased after pharmacological transporter blockade (Huang et al., 2004). To what extent synaptic crosstalk plays a role for low-affinity, post-synaptic AMPA receptor function is not fully explored. The net effect of the GLT-1 inhibitor DHK on AMPA receptor-mediated EPSCs varies between small decrease and no effect (Asztely et al., 1997 and references therein). Similarly, AMPA receptor-mediated EPSCs recorded from CA1 pyramidal cells were not affected by transporter blockade using TBOA, irrespective of whether these were action-potential independent EPSCs (“minis”) or evoked by minimal-stimulation (Zheng et al., 2008). This effectively rules out a role of glutamate transporters in controlling AMPA receptor activation at the releasing synapse. However, it does not exclude that: (1) glutamate may reach AMPA receptors at nearby synapses of other neurons in the densely packed neuropil (not recorded from); and (2) that astroglial transporters control such AMPA receptor-dependent crosstalk. The latter scenario is hinted at by our previous experimental findings. The inhibition of astroglial metabolism using the gliotoxin fluoroacetate (FAC) is likely to impair astroglial glutamate uptake (Karus et al., 2015). At the same time, this treatment significantly increased the predominantly AMPA receptor-mediated field EPSP slope, a measurement reflecting all AMPA receptor-activity in the neuropil, by about 20% (Henneberger et al., 2010b). However, a direct measurement of the range of action of glutamate and its dependence on astroglial transporters is still missing.

It should be noted here that the often used broad-spectrum glutamate transporter inhibitors TBOA and TFB-TBOA (Shimamoto et al., 1998) do not selectively inhibit astroglial transporters but dose-dependently inhibit GLT-1 (expressed mostly but not exclusively by astrocytes in the hippocampus Mennerick et al., 1998; Danbolt, 2001), the astroglial GLAST and the neuronally-expressed EAAC1 (Shigeri et al., 2004). However, the volume density of EAAC1 (molecules/μm^3^) is two orders of magnitude lower than GLT-1 in the CA1 *stratum radiatum*, which raises the question to what extent EAAC1 contributes specifically to glutamate clearance (Holmseth et al., 2012; but see Scimemi et al., 2009).

In summary, experimental evidence and modeling studies both support a role of astroglial glutamate uptake in constraining the invasion of extrasynaptic space by synaptically released glutamate and thereby the activation of high-affinity glutamate receptors beyond the active synapse.

### The Role of Perisynaptic Astroglial Structure for Glutamate Clearance

It is intuitive that the positioning and density of astroglial glutamate transporters in the perisynaptic space determines how far glutamate diffuses into perisynaptic or extrasynaptic ECS before it binds to astrocytic glutamate transporters. Therefore, the spatial arrangement of PAPs relative to the synapse (for instance distance or astrocyte surface per volume) is expected to shape perisynaptic glutamate signaling as much as the transporter density in these PAPs.

Interestingly, PAPs are capable of local protein synthesis and both GLAST and GLT-1 transcripts are enriched in PAPs (Sakers et al., 2017). Insertion of these transporters into the astroglial cell membrane and their internalization are regulated processes, which can depend on astroglial calcium levels (see “Astrocyte Calcium Signaling” section for a discussion of experimental evidence). In addition, cell surface expression of GLT-1 is regulated by protein kinase C activity (Kalandadze et al., 2002), sumoylation (in cultured astrocytes; Foran et al., 2014) and ubiquitination (in HEK293 cells; Ibáñez et al., 2016). Once inserted in the membrane, glutamate transporters are mobile in the astroglial membrane as revealed by single molecule tracking techniques (Murphy-Royal et al., 2015). The mobility of GLT-1 on the astroglial cell membrane depends on neuronal activity and the transporter activity itself in dissociated and slice cultures (Murphy-Royal et al., 2015; Al Awabdh et al., 2016). Interestingly, diffusion of GLT-1 in membranes slows down near synapses and cross-linking of GLT-1, which reduced its mobility, was shown to affect synaptic transmission (Murphy-Royal et al., 2015). Together these studies indicate that the transporter density in astroglial membranes is tightly regulated and tuned by neuronal activity.

Together with the transporter density, the abundance of astroglial processes and membranes in the vicinity of a synapse (or in other words the exact arrangement and structure of PAPs) determines the total number of available transporters. As a consequence, changes of astrocytic morphology that modify the spatial relationship between synapses and PAPs will affect how efficiently or rapidly glutamate is captured by astrocytic transporters and how likely it is for glutamate to escape into the peri- and extrasynaptic space (Figure 3C). Pioneering work on this interplay between astrocyte morphology and synapse function was performed using the oxytocinergic system of the supraoptic nucleus as a model system. In this brain region, astrocyte processes less efficiently separate neurons during lactation, which leads to the increased occurrence of juxta-positioned neuronal membrane profiles (Theodosis and Poulain, 1993). Importantly, this reduction of astroglial coverage of neurons during lactation is associated with a reduced efficiency of glutamate clearance and changes in the control of synaptic transmission by presynaptic metabotropic glutamate receptors (Oliet et al., 2001). Thus, changes of astrocyte structure and therefore the spatial configuration of the ECS surrounding neurons and nearby astrocytes determines the extracellular dynamics of synaptically-released glutamate in the supraoptic nucleus. In the following, we discuss the experimental evidence implying that glutamate clearance in the hippocampus could also depend on astroglial morphology and its changes.

Ventura and Harris reported in 1999 that only about half of the glutamatergic synapses in the CA1 *stratum radiatum* have astroglial processes directly apposed to them (Figure 3D), which in each case covered again only about half of the synaptic interface (Ventura and Harris, 1999). The efficiency of local glutamate clearance by astroglia is, therefore, expected to vary considerably from synapse to synapse. This observation raised the question, which synapses are particularly well-covered by astroglial transporters and what the functional relevance of the potential heterogeneity may be. Serial-section EM of astrocyte fragments in the dentate gyrus followed by their 3D-reconstruction along with nearby synapses provided some insights into this issue (Medvedev et al., 2014). It was found for instance that the diffusion-weighted average distance from the synapse to the surrounding astroglial processes was significantly smaller at thin synapses compared to larger, mushroom-type synapses (Figures 3E–G). This suggests that the size of the postsynaptic spine and therefore presumably synaptic strength (Schikorski and Stevens, 1997) are inversely correlated with local astroglial glutamate clearance. A testable hypothesis is therefore that larger synapses are associated with a lower efficiency of local astroglial glutamate clearance and that the glutamate escape into peri- and extrasynaptic space is more prominent. Such scenarios would be interesting to explore because spine size plasticity is tightly associated with synaptic plasticity (Bourne and Harris, 2007). It should be noted here that astrocyte morphologies also vary widely within and across brain regions (Matyash and Kettenmann, 2010; Anders et al., 2014) and therefore such relationships between astrocyte morphology and local abundance of astroglial glutamate transporters could be as heterogeneous.

Similar to the supraoptic nucleus, the morphology of astrocytes is not static in the hippocampus. Time lapse imaging of fluorescently-labeled astrocytes revealed a surprisingly high motility of astrocyte processes on a time scale of minutes in organotypic hippocampal slices cultures (Haber et al., 2006), which rapidly changed the relative positions to neuronal spines. A well-studied and potent trigger of PAP restructuring is the induction of synaptic plasticity (Wenzel et al., 1991; Lushnikova et al., 2009; Henneberger et al., 2010a; Bernardinelli et al., 2014; Perez-Alvarez et al., 2014). Plasticity-associated changes of PAP structure and motility have been shown to determine spine fate (Bernardinelli et al., 2014) and to affect astroglial control of presynaptic release (Perez-Alvarez et al., 2014). However, it is currently unclear if such rapid dynamic changes of PAP structure have an impact on astroglial glutamate uptake and synaptic crosstalk. Indirect supporting evidence is provided by elegant experiments using connexin 30 knockout mice in which PAPs invade the synaptic cleft and impair synaptic transmission and plasticity in a transporter-dependent manner (Pannasch et al., 2014). One potential experimental strategy to establish the effect of rapid changes of PAP/spine spatial relationships on glutamate clearance and synaptic crosstalk is to induce defined and isolated changes of PAP morphology. This could be done by targeting the signaling cascades that govern astrocyte morphology (Zeug et al., 2017) while monitoring glutamate signaling and crosstalk.

## Conclusion

Excitatory glutamatergic transmission between neurons in the hippocampus cannot be separated functionally from the action of synaptically-released glutamate onto neighboring astrocytes. Recent advances in technical approaches such as genetically-targeted stimulation of specific cell types and single cells, genetically-encoded indicators and super-resolution imaging have shed more light on this intricate relationship. On the one hand side, astrocytes express glutamate receptors that can trigger intracellular signaling cascades. On the other hand, astrocytic transporters bind glutamate with high affinity, which rapidly buffers the glutamate released from presynaptic terminals thereby shaping extracellular glutamate concentrations transients and thus modifying the activation of receptors on postsynaptic neurons. Once taken up, astrocytes may use glutamate to fuel their own metabolite needs, but also convert it to glutamine and cycle it back to neurons. The exact temporal and spatial dynamics of this interplay between neurons and astrocytes are just starting to emerge. Recent studies suggest that astrocytes not only undergo slow changes in their shape and characteristics in response to pathological events. Moreover, there is evidence that astrocyte perisynaptic processes might undergo rapid activity-dependent structural changes at the sub-micron scale that will influence their glutamate clearance and thus, the functional properties of glutamatergic synapses. Future work will hopefully provide new and exciting insights into this dynamic aspect of neuron-glia interaction, which is needed to fully understand the function of glutamatergic synapses in the hippocampus and in other regions of the brain.

## Author Contributions

All authors contributed to the conception and drafting of the work, revised it critically for important intellectual content and approved the final version to be published.

## Conflict of Interest Statement

The authors declare that the research was conducted in the absence of any commercial or financial relationships that could be construed as a potential conflict of interest.
